# 6′-(1,3-Diphenyl-1*H*-pyrazol-4-yl)-7′-(1*H*-indol-3-ylcarbon­yl)-2-oxo-1-(prop-2-en-1-yl)-5′,6′,7′,7a’-tetra­hydro-1′*H*-spiro­[indoline-3,5′-pyrrolo­[1,2-*c*][1,3]thia­zole]-7′-carbo­nitrile

**DOI:** 10.1107/S1600536813011513

**Published:** 2013-05-11

**Authors:** Piskala Subburaman Kannan, Devaraj Kathirvelan, Boreddy Siva Rami Reddy, Elumalai Govindan, Arunachalathevar SubbiahPandi

**Affiliations:** a Department of Physics, S.M.K. Fomra Institute of Technology, Thaiyur, Chennai 603 103, India; b Industrial Chemistry Laboratory, Central Leather Research Institute, Adyar, Chennai 600 020, India; c Department of Physics, Presidency College (Autonomous), Chennai 600 005, India

## Abstract

In the title compound, C_41_H_32_N_6_O_2_S, the pyrrolo­thia­zole ring system is folded about the bridging N—C bond. The thia­zolidine and pyrrolidine rings adopt envelope (with the fused C atom as the flap) and twisted conformations, respectively. The two phenyl rings attached to the pyrazole ring are twisted from the plane of the latter by 6.8 (1) and 52.8 (1)°. The allyl group is disordered over two conformations in a 0.805 (6):0.195 (6) ratio. In the crystal, pairs of N—H⋯O hydrogen bonds link the mol­ecules into centrosymmetric dimers.

## Related literature

For the biological activity of spiro­heterocycles, see: Kilonda *et al.* (1995 ▶); Ferguson *et al.* (2005 ▶). For related structures, see: Jagadeesan *et al.* (2012*a* ▶,*b* ▶). For ring conformations, see: Cremer & Pople (1975 ▶).
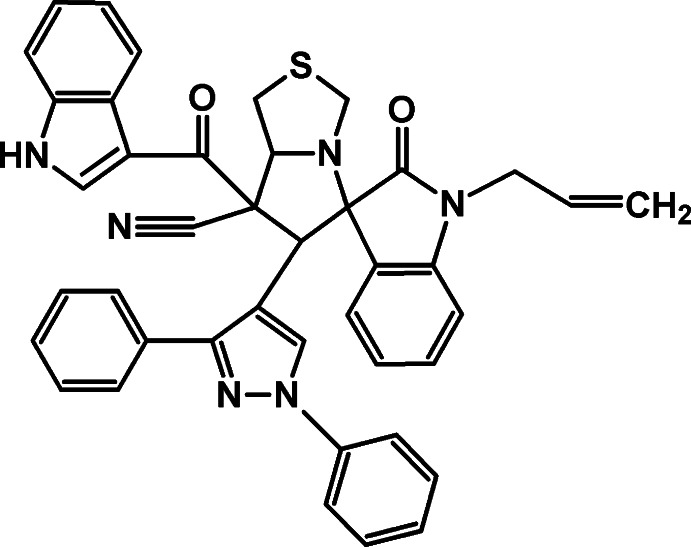


## Experimental

### 

#### Crystal data

C_41_H_32_N_6_O_2_S
*M* *_r_* = 672.80
Monoclinic, 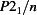

*a* = 10.7396 (10) Å
*b* = 16.3471 (14) Å
*c* = 20.112 (2) Å
β = 105.485 (4)°
*V* = 3402.7 (6) Å^3^
*Z* = 4
Mo *K*α radiation
μ = 0.14 mm^−1^
*T* = 293 K
0.30 × 0.25 × 0.20 mm

#### Data collection

Bruker SMART APEXII area-detector diffractometer
Absorption correction: multi-scan (*SADABS*; Bruker, 2008 ▶) *T*_min_ = 0.958, *T*_max_ = 0.972
25634 measured reflections
5983 independent reflections
3281 reflections with *I* > 2σ(*I*)
*R*_int_ = 0.049

#### Refinement

*R*[*F* ^2^ > 2σ(*F* ^2^)] = 0.049
*wR*(*F* ^2^) = 0.146
*S* = 1.06
5983 reflections
483 parameters
48 restraints
H atoms treated by a mixture of independent and constrained refinement
Δρ_max_ = 0.40 e Å^−3^
Δρ_min_ = −0.30 e Å^−3^

### 

Data collection: *APEX2* (Bruker, 2008 ▶); cell refinement: *SAINT* (Bruker, 2008 ▶); data reduction: *SAINT*; program(s) used to solve structure: *SHELXS97* (Sheldrick, 2008 ▶); program(s) used to refine structure: *SHELXL97* (Sheldrick, 2008 ▶); molecular graphics: *ORTEP-3 for Windows* (Farrugia, 2012 ▶); software used to prepare material for publication: *SHELXL97* and *PLATON* (Spek, 2009 ▶).

## Supplementary Material

Crystal structure: contains datablock(s) global, I. DOI: 10.1107/S1600536813011513/cv5404sup1.cif

Structure factors: contains datablock(s) I. DOI: 10.1107/S1600536813011513/cv5404Isup2.hkl

Supplementary material file. DOI: 10.1107/S1600536813011513/cv5404Isup3.cml

Additional supplementary materials:  crystallographic information; 3D view; checkCIF report

## Figures and Tables

**Table 1:** Hydrogen-bond geometry (Å, °)

*D*—H⋯*A*	*D*—H	H⋯*A*	*D*⋯*A*	*D*—H⋯*A*
N5—H5*A*⋯O1^i^	0.92 (4)	1.92 (4)	2.822 (4)	166 (3)

Symmetry code: (i) 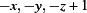.

**Fig. 1.**
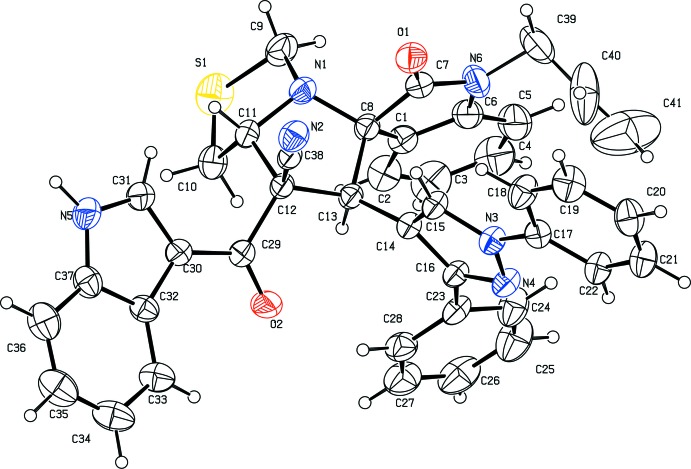
The molecular structure of (I) showing the atomic numbering and 30% probabilty displacement ellipsoids. For the disordered allyl group, only major part is shown.

## supplementary crystallographic information

### Comment

The design and synthesis of glycospiroheterocycles are important in view of their biological profile against viruses, bacteria, and cancer cells (Ferguson *et al.*, 2005). Pyrrolidines and pyrroles are common structural motifs in drugs and drug candidates owing to their ability to act as selective glycosidase inhibitors, which are used in the treatment of diabetes, cancer, malaria and viral infections, including AIDS (Kilonda *et al.*, 1995). Herewith we present the title compound (I), obtained in our group and containing glycospiroheterocycles.

In (I) (Fig. 1), the indole ring system(C1-C8/N6) is essentially planar (r.m.s.deviation = -0.064 Å ). The pyrollidine ring C8/C11-C13/N1) forms dihedral angle of 60.3 (1) with thiazole ring (C9-C11/N1/S1). The thiazole ring adopts an envelope conformation on C11 atom, with the puckering parameters of q_2_ = 0.363 (3) Å, φ = 285.6 (5)° (Cremer & Pople, 1975). The pyrollidine ring adopts twisted conformation on C11-C12 atom, with the puckering parameters of q_2_ = 0.367 (3) Å, φ = 301.1 (5)°. The allyl group is disordered over two conformations in a ratio 0.805 (6):0.195 (6). The crystal packing features are intermolecular N—H···O hydrogen bonds (Table 1), which link the molecules into centrosymmetric dimers.

### Experimental

A mixture of allyl Isatin (1 mmol), thiaproline(1.1 mmol) and (2E)-3-(1,3- diphenyl-1H-pyrazol-4-yl)-2-[(E)-(1H-indol-3-yl) carbonyl] prop-2-enenitrile (1.3 mmol) were refluxed in ethanol (5 mL) for about 3 h. Completion of the reaction was evidenced by TLC analysis. After completion of the reaction, the reaction mixture was poured into ice-water, the resulting solid was filtered off and purified by column chromatography using ethyl acetate/Ether (6:4) as an eluent to afford pure spirooxindole.The block shaped single crystals of the title compound suitable for X-ray diffraction were obtained from solution of ethyl acetate/Ether (6:4) by slow evaportion at room temperature.

### Refinement

Amino H atom was located on difference map and isotropically refined. C-bound H atoms were positioned geometrically, with C—H = 0.93 - 0.98 Å, and allowed to ride on their parent atoms, with *U*_iso_(H) = 1.2–1.5 *U*_eq_(C). The positions of methyl hydrogens were optimized rotationally.

### Crystal data

**Table:**

C_41_H_32_N_6_O_2_S	*F*(000) = 1408
*M**_r_* = 672.80	*D*_x_ = 1.313 Mg m^−^^3^
Monoclinic, *P*2_1_/*n*	Mo *K*α radiation, λ = 0.71073 Å
Hall symbol: -P 2yn	Cell parameters from 5983 reflections
*a* = 10.7396 (10) Å	θ = 1.6–25.0°
*b* = 16.3471 (14) Å	µ = 0.14 mm^−^^1^
*c* = 20.112 (2) Å	*T* = 293 K
β = 105.485 (4)°	Block, colourless
*V* = 3402.7 (6) Å^3^	0.30 × 0.25 × 0.20 mm
*Z* = 4	

### Data collection

**Table:**

Bruker SMART APEXII area-detector diffractometer	5983 independent reflections
Radiation source: fine-focus sealed tube	3281 reflections with *I* > 2σ(*I*)
Graphite monochromator	*R*_int_ = 0.049
ω and φ scans	θ_max_ = 25.0°, θ_min_ = 1.6°
Absorption correction: multi-scan (*SADABS*; Bruker, 2008)	*h* = −12→12
*T*_min_ = 0.958, *T*_max_ = 0.972	*k* = −19→19
25634 measured reflections	*l* = −23→23

### Refinement

**Table:**

Refinement on *F*^2^	Primary atom site location: structure-invariant direct methods
Least-squares matrix: full	Secondary atom site location: difference Fourier map
*R*[*F*^2^ > 2σ(*F*^2^)] = 0.049	Hydrogen site location: inferred from neighbouring sites
*wR*(*F*^2^) = 0.146	H atoms treated by a mixture of independent and constrained refinement
*S* = 1.06	*w* = 1/[σ^2^(*F*_o_^2^) + (0.0428*P*)^2^ + 2.1778*P*] where *P* = (*F*_o_^2^ + 2*F*_c_^2^)/3
5983 reflections	(Δ/σ)_max_ < 0.001
483 parameters	Δρ_max_ = 0.40 e Å^−^^3^
48 restraints	Δρ_min_ = −0.30 e Å^−^^3^

### Special details

**Table:**

Geometry. All esds (except the esd in the dihedral angle between two l.s. planes) are estimated using the full covariance matrix. The cell esds are taken into account individually in the estimation of esds in distances, angles and torsion angles; correlations between esds in cell parameters are only used when they are defined by crystal symmetry. An approximate (isotropic) treatment of cell esds is used for estimating esds involving l.s. planes.
Refinement. Refinement of F^2^ against ALL reflections. The weighted R-factor wR and goodness of fit S are based on F^2^, conventional R-factors R are based on F, with F set to zero for negative F^2^. The threshold expression of F^2^ > 2sigma(F^2^) is used only for calculating R-factors(gt) etc. and is not relevant to the choice of reflections for refinement. R-factors based on F^2^ are statistically about twice as large as those based on F, and R- factors based on ALL data will be even larger.

### Fractional atomic coordinates and isotropic or equivalent isotropic displacement parameters (Å^2^)

**Table:**

	*x*	*y*	*z*	*U*_iso_*/*U*_eq_	Occ. (<1)
C1	0.4320 (3)	0.18987 (19)	0.66619 (17)	0.0547 (8)	
C2	0.5399 (4)	0.1990 (2)	0.6438 (2)	0.0728 (11)	
H2	0.5475	0.1718	0.6045	0.087*	
C3	0.6388 (4)	0.2494 (3)	0.6804 (3)	0.0938 (14)	
H3	0.7128	0.2561	0.6653	0.113*	
C4	0.6281 (5)	0.2889 (3)	0.7378 (3)	0.0995 (15)	
H4	0.6957	0.3221	0.7617	0.119*	
C5	0.5211 (4)	0.2813 (3)	0.7615 (2)	0.0883 (13)	
H5	0.5141	0.3087	0.8009	0.106*	
C6	0.4237 (4)	0.2312 (2)	0.72452 (19)	0.0652 (10)	
C7	0.2358 (3)	0.1605 (2)	0.69221 (17)	0.0563 (9)	
C8	0.3155 (3)	0.13548 (19)	0.64258 (15)	0.0486 (8)	
C9	0.2343 (4)	0.2121 (2)	0.53083 (18)	0.0754 (11)	
H10A	0.2792	0.2563	0.5597	0.090*	
H10B	0.1472	0.2301	0.5084	0.090*	
C10	0.3350 (3)	0.0798 (2)	0.49222 (17)	0.0665 (10)	
H11A	0.3173	0.0440	0.4523	0.080*	
H11B	0.4214	0.0687	0.5209	0.080*	
C11	0.2359 (3)	0.06800 (19)	0.53247 (14)	0.0497 (8)	
H12	0.1510	0.0583	0.5003	0.060*	
C12	0.2648 (3)	−0.00061 (18)	0.58780 (14)	0.0444 (7)	
C13	0.3580 (3)	0.04386 (17)	0.64941 (13)	0.0435 (7)	
H14	0.4431	0.0424	0.6402	0.052*	
C14	0.3774 (3)	0.00947 (18)	0.72085 (14)	0.0458 (7)	
C15	0.2870 (3)	−0.01129 (19)	0.75412 (15)	0.0507 (8)	
H16	0.1981	−0.0129	0.7348	0.061*	
C16	0.4967 (3)	0.00417 (19)	0.77203 (15)	0.0474 (8)	
C17	0.2943 (3)	−0.04999 (18)	0.87456 (15)	0.0488 (8)	
C18	0.1634 (3)	−0.0618 (2)	0.86197 (17)	0.0664 (10)	
H19	0.1106	−0.0585	0.8171	0.080*	
C19	0.1106 (4)	−0.0784 (2)	0.9156 (2)	0.0737 (11)	
H20	0.0219	−0.0861	0.9070	0.088*	
C20	0.1878 (4)	−0.0837 (2)	0.98147 (19)	0.0699 (10)	
H21	0.1520	−0.0946	1.0179	0.084*	
C21	0.3172 (4)	−0.0731 (2)	0.99347 (17)	0.0690 (10)	
H22	0.3698	−0.0770	1.0383	0.083*	
C22	0.3720 (3)	−0.0566 (2)	0.94067 (15)	0.0583 (9)	
H23	0.4609	−0.0499	0.9496	0.070*	
C23	0.6270 (3)	0.0243 (2)	0.76733 (16)	0.0555 (9)	
C24	0.7013 (4)	0.0793 (3)	0.81318 (19)	0.0784 (11)	
H25	0.6691	0.1022	0.8475	0.094*	
C25	0.8227 (4)	0.1005 (3)	0.8086 (2)	0.0993 (15)	
H26	0.8719	0.1377	0.8398	0.119*	
C26	0.8714 (4)	0.0672 (3)	0.7582 (3)	0.0971 (15)	
H27	0.9536	0.0815	0.7552	0.117*	
C27	0.7988 (4)	0.0127 (3)	0.7123 (2)	0.0863 (13)	
H28	0.8314	−0.0096	0.6778	0.104*	
C28	0.6776 (3)	−0.0092 (2)	0.71694 (18)	0.0686 (10)	
H29	0.6292	−0.0468	0.6859	0.082*	
C29	0.3280 (3)	−0.07809 (19)	0.56533 (15)	0.0512 (8)	
C30	0.2501 (3)	−0.12852 (19)	0.51135 (16)	0.0517 (8)	
C31	0.1356 (3)	−0.1070 (2)	0.46489 (17)	0.0636 (9)	
H32	0.0976	−0.0556	0.4623	0.076*	
C32	0.2721 (3)	−0.21251 (19)	0.49676 (16)	0.0519 (8)	
C33	0.3645 (3)	−0.2707 (2)	0.5256 (2)	0.0683 (10)	
H38	0.4361	−0.2570	0.5614	0.082*	
C34	0.3477 (4)	−0.3495 (2)	0.5000 (3)	0.0857 (12)	
H37	0.4091	−0.3891	0.5190	0.103*	
C35	0.2419 (5)	−0.3710 (3)	0.4468 (3)	0.0896 (13)	
H36	0.2336	−0.4247	0.4312	0.108*	
C36	0.1504 (4)	−0.3159 (2)	0.4171 (2)	0.0781 (11)	
H35	0.0799	−0.3304	0.3809	0.094*	
C37	0.1658 (3)	−0.2367 (2)	0.44285 (18)	0.0609 (9)	
C38	0.1446 (3)	−0.02590 (19)	0.60293 (14)	0.0464 (8)	
N1	0.2303 (2)	0.14115 (16)	0.57223 (13)	0.0539 (7)	
N2	0.0520 (3)	−0.04893 (18)	0.61364 (14)	0.0620 (8)	
N3	0.3494 (2)	−0.02919 (15)	0.82025 (12)	0.0496 (6)	
N4	0.4784 (2)	−0.01928 (16)	0.83205 (12)	0.0532 (7)	
N5	0.0868 (3)	−0.1700 (2)	0.42411 (16)	0.0692 (9)	
N6	0.3063 (3)	0.2139 (2)	0.73847 (15)	0.0736 (9)	
O1	0.1263 (2)	0.13763 (15)	0.68978 (12)	0.0691 (7)	
O2	0.4379 (2)	−0.09462 (15)	0.59576 (13)	0.0772 (8)	
S1	0.31860 (13)	0.18539 (7)	0.46628 (6)	0.0945 (4)	
C39	0.2629 (10)	0.2521 (5)	0.7937 (4)	0.107 (3)	0.805 (6)
H39A	0.1693	0.2519	0.7825	0.128*	0.805 (6)
H39B	0.2925	0.3084	0.7999	0.128*	0.805 (6)
C40	0.3168 (9)	0.2052 (6)	0.8568 (3)	0.152 (4)	0.805 (6)
H40	0.2549	0.1675	0.8617	0.183*	0.805 (6)
C41	0.4063 (9)	0.1972 (8)	0.9026 (6)	0.229 (6)	0.805 (6)
H41A	0.4784	0.2301	0.9063	0.275*	0.805 (6)
H41B	0.4066	0.1578	0.9360	0.275*	0.805 (6)
C39'	0.263 (5)	0.227 (3)	0.8005 (14)	0.125 (8)	0.195 (6)
H39C	0.3337	0.2456	0.8381	0.150*	0.195 (6)
H39D	0.2294	0.1763	0.8143	0.150*	0.195 (6)
C40'	0.161 (3)	0.2890 (18)	0.7835 (14)	0.113 (7)	0.195 (6)
H40'	0.1931	0.3408	0.7790	0.136*	0.195 (6)
C41'	0.035 (3)	0.2896 (15)	0.7732 (13)	0.119 (9)	0.195 (6)
H41C	−0.0086	0.2413	0.7763	0.143*	0.195 (6)
H41D	−0.0104	0.3384	0.7627	0.143*	0.195 (6)
H5A	0.010 (4)	−0.166 (2)	0.390 (2)	0.103 (15)*	

### Atomic displacement parameters (Å^2^)

**Table:**

	*U*^11^	*U*^22^	*U*^33^	*U*^12^	*U*^13^	*U*^23^
C1	0.054 (2)	0.049 (2)	0.059 (2)	−0.0032 (16)	0.0118 (17)	0.0035 (17)
C2	0.072 (3)	0.072 (3)	0.074 (2)	−0.016 (2)	0.019 (2)	0.005 (2)
C3	0.077 (3)	0.094 (3)	0.108 (4)	−0.031 (3)	0.021 (3)	0.008 (3)
C4	0.080 (3)	0.083 (3)	0.123 (4)	−0.029 (3)	0.006 (3)	−0.013 (3)
C5	0.084 (3)	0.076 (3)	0.094 (3)	−0.008 (2)	0.006 (3)	−0.024 (2)
C6	0.058 (2)	0.057 (2)	0.073 (2)	0.0010 (18)	0.0041 (19)	−0.011 (2)
C7	0.050 (2)	0.061 (2)	0.054 (2)	0.0093 (18)	0.0082 (17)	−0.0019 (17)
C8	0.0474 (18)	0.0513 (19)	0.0440 (17)	−0.0004 (15)	0.0069 (15)	−0.0005 (15)
C9	0.094 (3)	0.061 (2)	0.062 (2)	0.003 (2)	0.006 (2)	0.0136 (19)
C10	0.076 (2)	0.075 (2)	0.052 (2)	−0.003 (2)	0.0224 (18)	0.0053 (18)
C11	0.0509 (19)	0.057 (2)	0.0380 (16)	0.0000 (15)	0.0054 (14)	0.0006 (15)
C12	0.0410 (18)	0.0521 (19)	0.0380 (16)	0.0016 (14)	0.0067 (13)	0.0011 (14)
C13	0.0393 (17)	0.0503 (19)	0.0387 (16)	0.0010 (14)	0.0068 (13)	0.0010 (14)
C14	0.0447 (18)	0.0496 (19)	0.0408 (16)	−0.0011 (15)	0.0071 (14)	0.0004 (14)
C15	0.0448 (18)	0.061 (2)	0.0414 (17)	−0.0014 (16)	0.0035 (14)	0.0038 (16)
C16	0.0428 (18)	0.058 (2)	0.0402 (17)	0.0016 (15)	0.0080 (14)	0.0072 (15)
C17	0.055 (2)	0.0459 (19)	0.0473 (18)	−0.0052 (15)	0.0164 (16)	0.0009 (15)
C18	0.056 (2)	0.089 (3)	0.052 (2)	−0.0110 (19)	0.0126 (17)	0.0085 (19)
C19	0.067 (2)	0.088 (3)	0.073 (3)	−0.015 (2)	0.031 (2)	0.004 (2)
C20	0.086 (3)	0.076 (3)	0.057 (2)	−0.014 (2)	0.034 (2)	0.002 (2)
C21	0.076 (3)	0.083 (3)	0.048 (2)	−0.012 (2)	0.0162 (19)	0.0013 (19)
C22	0.057 (2)	0.071 (2)	0.0456 (19)	−0.0082 (17)	0.0118 (16)	0.0005 (17)
C23	0.0428 (19)	0.075 (2)	0.0446 (18)	0.0009 (17)	0.0035 (15)	0.0145 (18)
C24	0.060 (2)	0.112 (3)	0.060 (2)	−0.021 (2)	0.0109 (19)	0.002 (2)
C25	0.063 (3)	0.144 (4)	0.085 (3)	−0.034 (3)	0.009 (2)	0.007 (3)
C26	0.047 (2)	0.144 (4)	0.100 (3)	−0.005 (3)	0.018 (3)	0.034 (3)
C27	0.057 (3)	0.121 (4)	0.084 (3)	0.011 (3)	0.025 (2)	0.015 (3)
C28	0.048 (2)	0.088 (3)	0.068 (2)	0.0096 (19)	0.0129 (18)	0.004 (2)
C29	0.048 (2)	0.055 (2)	0.0472 (18)	0.0025 (16)	0.0078 (15)	−0.0023 (16)
C30	0.050 (2)	0.052 (2)	0.0503 (19)	0.0031 (16)	0.0086 (16)	−0.0018 (16)
C31	0.065 (2)	0.058 (2)	0.060 (2)	0.0074 (18)	0.0022 (18)	−0.0090 (19)
C32	0.0477 (19)	0.049 (2)	0.061 (2)	−0.0011 (16)	0.0181 (16)	−0.0033 (17)
C33	0.058 (2)	0.058 (2)	0.090 (3)	−0.0006 (19)	0.021 (2)	0.000 (2)
C34	0.081 (3)	0.054 (3)	0.128 (4)	0.005 (2)	0.039 (3)	0.001 (3)
C35	0.098 (4)	0.052 (3)	0.124 (4)	−0.018 (3)	0.041 (3)	−0.017 (3)
C36	0.085 (3)	0.065 (3)	0.086 (3)	−0.022 (2)	0.025 (2)	−0.016 (2)
C37	0.061 (2)	0.059 (2)	0.064 (2)	−0.0093 (19)	0.0185 (18)	−0.0118 (19)
C38	0.046 (2)	0.0482 (19)	0.0394 (17)	0.0018 (16)	0.0014 (14)	0.0014 (14)
N1	0.0606 (17)	0.0504 (17)	0.0463 (15)	0.0052 (13)	0.0064 (13)	0.0052 (14)
N2	0.0504 (18)	0.072 (2)	0.0608 (17)	−0.0055 (15)	0.0096 (14)	0.0013 (15)
N3	0.0464 (16)	0.0569 (16)	0.0442 (15)	−0.0036 (13)	0.0098 (12)	0.0047 (13)
N4	0.0437 (16)	0.0666 (18)	0.0463 (15)	−0.0016 (13)	0.0067 (12)	0.0095 (13)
N5	0.065 (2)	0.070 (2)	0.0615 (19)	−0.0029 (18)	−0.0039 (16)	−0.0120 (17)
N6	0.062 (2)	0.089 (2)	0.067 (2)	0.0041 (17)	0.0126 (16)	−0.0324 (18)
O1	0.0481 (15)	0.0919 (18)	0.0664 (15)	0.0064 (13)	0.0135 (12)	−0.0044 (13)
O2	0.0530 (15)	0.0819 (18)	0.0825 (17)	0.0177 (13)	−0.0065 (13)	−0.0255 (14)
S1	0.1178 (10)	0.0885 (8)	0.0832 (7)	−0.0166 (7)	0.0373 (7)	0.0253 (6)
C39	0.093 (4)	0.115 (6)	0.115 (5)	0.011 (5)	0.031 (4)	−0.059 (4)
C40	0.152 (7)	0.253 (9)	0.067 (4)	−0.062 (7)	0.054 (4)	−0.047 (6)
C41	0.163 (10)	0.305 (14)	0.252 (13)	0.092 (9)	0.115 (9)	0.137 (11)
C39'	0.120 (12)	0.159 (14)	0.086 (11)	0.000 (12)	0.012 (11)	−0.050 (12)
C40'	0.106 (13)	0.118 (13)	0.121 (12)	−0.002 (12)	0.038 (12)	−0.032 (11)
C41'	0.18 (2)	0.067 (15)	0.111 (18)	0.011 (16)	0.033 (18)	−0.005 (13)

### Geometric parameters (Å, º)

**Table:**

C1—C2	1.358 (5)	C21—H22	0.9300
C1—C6	1.378 (5)	C22—H23	0.9300
C1—C8	1.505 (4)	C23—C24	1.379 (5)
C2—C3	1.390 (5)	C23—C28	1.384 (5)
C2—H2	0.9300	C24—C25	1.375 (5)
C3—C4	1.354 (6)	C24—H25	0.9300
C3—H3	0.9300	C25—C26	1.373 (6)
C4—C5	1.362 (6)	C25—H26	0.9300
C4—H4	0.9300	C26—C27	1.366 (6)
C5—C6	1.380 (5)	C26—H27	0.9300
C5—H5	0.9300	C27—C28	1.378 (5)
C6—N6	1.392 (4)	C27—H28	0.9300
C7—O1	1.222 (4)	C28—H29	0.9300
C7—N6	1.351 (4)	C29—O2	1.207 (3)
C7—C8	1.533 (4)	C29—C30	1.440 (4)
C8—N1	1.468 (4)	C30—C31	1.376 (4)
C8—C13	1.561 (4)	C30—C32	1.437 (4)
C9—N1	1.435 (4)	C31—N5	1.333 (4)
C9—S1	1.823 (4)	C31—H32	0.9300
C9—H10A	0.9700	C32—C33	1.385 (4)
C9—H10B	0.9700	C32—C37	1.405 (4)
C10—C11	1.512 (4)	C33—C34	1.381 (5)
C10—S1	1.798 (4)	C33—H38	0.9300
C10—H11A	0.9700	C34—C35	1.381 (6)
C10—H11B	0.9700	C34—H37	0.9300
C11—N1	1.449 (4)	C35—C36	1.349 (5)
C11—C12	1.552 (4)	C35—H36	0.9300
C11—H12	0.9800	C36—C37	1.388 (5)
C12—C38	1.462 (4)	C36—H35	0.9300
C12—C13	1.550 (4)	C37—N5	1.372 (5)
C12—C29	1.560 (4)	C38—N2	1.137 (4)
C13—C14	1.505 (4)	N3—N4	1.352 (3)
C13—H14	0.9800	N5—H5A	0.92 (4)
C14—C15	1.361 (4)	N6—C39	1.454 (6)
C14—C16	1.416 (4)	N6—C39'	1.460 (11)
C15—N3	1.352 (4)	C39—C40	1.465 (8)
C15—H16	0.9300	C39—H39A	0.9700
C16—N4	1.330 (4)	C39—H39B	0.9700
C16—C23	1.465 (4)	C40—C41	1.147 (8)
C17—C22	1.372 (4)	C40—H40	0.9300
C17—C18	1.373 (4)	C41—H41A	0.9300
C17—N3	1.416 (4)	C41—H41B	0.9300
C18—C19	1.374 (4)	C39'—C40'	1.466 (11)
C18—H19	0.9300	C39'—H39C	0.9700
C19—C20	1.365 (5)	C39'—H39D	0.9700
C19—H20	0.9300	C40'—C41'	1.307 (10)
C20—C21	1.357 (5)	C40'—H40'	0.9300
C20—H21	0.9300	C41'—H41C	0.9300
C21—C22	1.371 (4)	C41'—H41D	0.9300
			
C2—C1—C6	118.9 (3)	C28—C23—C16	121.8 (3)
C2—C1—C8	132.2 (3)	C25—C24—C23	120.6 (4)
C6—C1—C8	108.7 (3)	C25—C24—H25	119.7
C1—C2—C3	119.3 (4)	C23—C24—H25	119.7
C1—C2—H2	120.4	C26—C25—C24	120.3 (4)
C3—C2—H2	120.4	C26—C25—H26	119.9
C4—C3—C2	120.5 (4)	C24—C25—H26	119.9
C4—C3—H3	119.7	C27—C26—C25	119.8 (4)
C2—C3—H3	119.7	C27—C26—H27	120.1
C3—C4—C5	121.7 (4)	C25—C26—H27	120.1
C3—C4—H4	119.1	C26—C27—C28	120.2 (4)
C5—C4—H4	119.1	C26—C27—H28	119.9
C4—C5—C6	117.1 (4)	C28—C27—H28	119.9
C4—C5—H5	121.5	C27—C28—C23	120.7 (4)
C6—C5—H5	121.5	C27—C28—H29	119.7
C1—C6—C5	122.5 (4)	C23—C28—H29	119.7
C1—C6—N6	109.9 (3)	O2—C29—C30	123.2 (3)
C5—C6—N6	127.6 (4)	O2—C29—C12	118.5 (3)
O1—C7—N6	125.5 (3)	C30—C29—C12	118.2 (3)
O1—C7—C8	126.2 (3)	C31—C30—C32	105.7 (3)
N6—C7—C8	108.3 (3)	C31—C30—C29	126.8 (3)
N1—C8—C1	121.2 (3)	C32—C30—C29	127.4 (3)
N1—C8—C7	107.4 (2)	N5—C31—C30	110.7 (3)
C1—C8—C7	101.5 (3)	N5—C31—H32	124.6
N1—C8—C13	103.7 (2)	C30—C31—H32	124.6
C1—C8—C13	109.9 (2)	C33—C32—C37	118.2 (3)
C7—C8—C13	113.5 (2)	C33—C32—C30	135.2 (3)
N1—C9—S1	108.5 (2)	C37—C32—C30	106.4 (3)
N1—C9—H10A	110.0	C34—C33—C32	118.5 (4)
S1—C9—H10A	110.0	C34—C33—H38	120.8
N1—C9—H10B	110.0	C32—C33—H38	120.8
S1—C9—H10B	110.0	C35—C34—C33	121.6 (4)
H10A—C9—H10B	108.4	C35—C34—H37	119.2
C11—C10—S1	104.4 (2)	C33—C34—H37	119.2
C11—C10—H11A	110.9	C36—C35—C34	121.7 (4)
S1—C10—H11A	110.9	C36—C35—H36	119.2
C11—C10—H11B	110.9	C34—C35—H36	119.2
S1—C10—H11B	110.9	C35—C36—C37	117.1 (4)
H11A—C10—H11B	108.9	C35—C36—H35	121.4
N1—C11—C10	109.2 (3)	C37—C36—H35	121.4
N1—C11—C12	103.3 (2)	N5—C37—C36	129.5 (4)
C10—C11—C12	116.2 (3)	N5—C37—C32	107.7 (3)
N1—C11—H12	109.3	C36—C37—C32	122.9 (4)
C10—C11—H12	109.3	N2—C38—C12	177.0 (3)
C12—C11—H12	109.3	C9—N1—C11	109.6 (2)
C38—C12—C13	112.6 (2)	C9—N1—C8	120.2 (3)
C38—C12—C11	109.6 (2)	C11—N1—C8	111.9 (2)
C13—C12—C11	101.4 (2)	N4—N3—C15	111.3 (2)
C38—C12—C29	107.9 (2)	N4—N3—C17	120.9 (2)
C13—C12—C29	112.0 (2)	C15—N3—C17	127.7 (3)
C11—C12—C29	113.5 (2)	C16—N4—N3	105.5 (2)
C14—C13—C12	118.9 (2)	C31—N5—C37	109.4 (3)
C14—C13—C8	113.9 (2)	C31—N5—H5A	122 (3)
C12—C13—C8	105.6 (2)	C37—N5—H5A	129 (3)
C14—C13—H14	105.8	C7—N6—C6	111.3 (3)
C12—C13—H14	105.8	C7—N6—C39	124.7 (6)
C8—C13—H14	105.8	C6—N6—C39	124.0 (6)
C15—C14—C16	104.7 (3)	C7—N6—C39'	116 (3)
C15—C14—C13	128.9 (3)	C6—N6—C39'	131 (2)
C16—C14—C13	125.8 (3)	C39—N6—C39'	17 (2)
N3—C15—C14	107.8 (3)	C10—S1—C9	92.97 (16)
N3—C15—H16	126.1	N6—C39—C40	107.7 (5)
C14—C15—H16	126.1	N6—C39—H39A	110.2
N4—C16—C14	110.7 (3)	C40—C39—H39A	110.2
N4—C16—C23	119.9 (3)	N6—C39—H39B	110.2
C14—C16—C23	129.4 (3)	C40—C39—H39B	110.2
C22—C17—C18	119.7 (3)	H39A—C39—H39B	108.5
C22—C17—N3	119.7 (3)	C41—C40—C39	143.9 (12)
C18—C17—N3	120.6 (3)	C41—C40—H40	108.0
C17—C18—C19	120.0 (3)	C39—C40—H40	108.0
C17—C18—H19	120.0	C40—C41—H41A	120.0
C19—C18—H19	120.0	C40—C41—H41B	120.0
C20—C19—C18	120.2 (4)	H41A—C41—H41B	120.0
C20—C19—H20	119.9	N6—C39'—C40'	107.0 (19)
C18—C19—H20	119.9	N6—C39'—H39C	110.3
C21—C20—C19	119.5 (3)	C40'—C39'—H39C	110.3
C21—C20—H21	120.2	N6—C39'—H39D	110.3
C19—C20—H21	120.2	C40'—C39'—H39D	110.3
C20—C21—C22	121.2 (3)	H39C—C39'—H39D	108.6
C20—C21—H22	119.4	C41'—C40'—C39'	136 (4)
C22—C21—H22	119.4	C41'—C40'—H40'	112.1
C21—C22—C17	119.4 (3)	C39'—C40'—H40'	112.1
C21—C22—H23	120.3	C40'—C41'—H41C	120.0
C17—C22—H23	120.3	C40'—C41'—H41D	120.0
C24—C23—C28	118.5 (3)	H41C—C41'—H41D	120.0
C24—C23—C16	119.7 (3)		
			
C6—C1—C2—C3	0.2 (5)	C11—C12—C29—O2	113.1 (3)
C8—C1—C2—C3	−173.9 (3)	C38—C12—C29—C30	51.9 (3)
C1—C2—C3—C4	0.2 (6)	C13—C12—C29—C30	176.3 (3)
C2—C3—C4—C5	−0.4 (7)	C11—C12—C29—C30	−69.7 (3)
C3—C4—C5—C6	0.2 (7)	O2—C29—C30—C31	−165.7 (3)
C2—C1—C6—C5	−0.4 (5)	C12—C29—C30—C31	17.3 (5)
C8—C1—C6—C5	175.0 (3)	O2—C29—C30—C32	17.4 (5)
C2—C1—C6—N6	179.8 (3)	C12—C29—C30—C32	−159.6 (3)
C8—C1—C6—N6	−4.8 (4)	C32—C30—C31—N5	0.2 (4)
C4—C5—C6—C1	0.2 (6)	C29—C30—C31—N5	−177.2 (3)
C4—C5—C6—N6	180.0 (4)	C31—C30—C32—C33	−176.8 (4)
C2—C1—C8—N1	−61.0 (5)	C29—C30—C32—C33	0.7 (6)
C6—C1—C8—N1	124.4 (3)	C31—C30—C32—C37	−1.6 (4)
C2—C1—C8—C7	−179.6 (4)	C29—C30—C32—C37	175.9 (3)
C6—C1—C8—C7	5.8 (3)	C37—C32—C33—C34	0.1 (5)
C2—C1—C8—C13	60.0 (4)	C30—C32—C33—C34	174.8 (4)
C6—C1—C8—C13	−114.6 (3)	C32—C33—C34—C35	0.0 (6)
O1—C7—C8—N1	45.5 (4)	C33—C34—C35—C36	0.5 (7)
N6—C7—C8—N1	−133.1 (3)	C34—C35—C36—C37	−0.9 (6)
O1—C7—C8—C1	173.6 (3)	C35—C36—C37—N5	−177.8 (4)
N6—C7—C8—C1	−5.0 (3)	C35—C36—C37—C32	1.0 (6)
O1—C7—C8—C13	−68.6 (4)	C33—C32—C37—N5	178.5 (3)
N6—C7—C8—C13	112.8 (3)	C30—C32—C37—N5	2.3 (4)
S1—C10—C11—N1	−37.1 (3)	C33—C32—C37—C36	−0.6 (5)
S1—C10—C11—C12	−153.4 (2)	C30—C32—C37—C36	−176.7 (3)
N1—C11—C12—C38	82.0 (3)	C13—C12—C38—N2	−130 (6)
C10—C11—C12—C38	−158.4 (3)	C11—C12—C38—N2	118 (6)
N1—C11—C12—C13	−37.1 (3)	C29—C12—C38—N2	−6 (6)
C10—C11—C12—C13	82.4 (3)	S1—C9—N1—C11	−26.7 (3)
N1—C11—C12—C29	−157.3 (2)	S1—C9—N1—C8	105.0 (3)
C10—C11—C12—C29	−37.8 (4)	C10—C11—N1—C9	42.3 (3)
C38—C12—C13—C14	43.6 (4)	C12—C11—N1—C9	166.6 (3)
C11—C12—C13—C14	160.6 (2)	C10—C11—N1—C8	−93.6 (3)
C29—C12—C13—C14	−78.1 (3)	C12—C11—N1—C8	30.7 (3)
C38—C12—C13—C8	−85.7 (3)	C1—C8—N1—C9	−17.2 (4)
C11—C12—C13—C8	31.3 (3)	C7—C8—N1—C9	98.4 (3)
C29—C12—C13—C8	152.6 (2)	C13—C8—N1—C9	−141.1 (3)
N1—C8—C13—C14	−146.4 (2)	C1—C8—N1—C11	113.4 (3)
C1—C8—C13—C14	82.7 (3)	C7—C8—N1—C11	−131.0 (3)
C7—C8—C13—C14	−30.1 (3)	C13—C8—N1—C11	−10.5 (3)
N1—C8—C13—C12	−14.1 (3)	C14—C15—N3—N4	1.2 (3)
C1—C8—C13—C12	−145.1 (2)	C14—C15—N3—C17	176.7 (3)
C7—C8—C13—C12	102.1 (3)	C22—C17—N3—N4	3.0 (4)
C12—C13—C14—C15	−51.9 (4)	C18—C17—N3—N4	−178.5 (3)
C8—C13—C14—C15	73.5 (4)	C22—C17—N3—C15	−172.0 (3)
C12—C13—C14—C16	139.0 (3)	C18—C17—N3—C15	6.4 (5)
C8—C13—C14—C16	−95.6 (3)	C14—C16—N4—N3	0.1 (3)
C16—C14—C15—N3	−1.1 (3)	C23—C16—N4—N3	177.1 (3)
C13—C14—C15—N3	−172.0 (3)	C15—N3—N4—C16	−0.8 (3)
C15—C14—C16—N4	0.7 (4)	C17—N3—N4—C16	−176.6 (3)
C13—C14—C16—N4	171.9 (3)	C30—C31—N5—C37	1.2 (4)
C15—C14—C16—C23	−176.0 (3)	C36—C37—N5—C31	176.7 (4)
C13—C14—C16—C23	−4.7 (5)	C32—C37—N5—C31	−2.2 (4)
C22—C17—C18—C19	1.3 (5)	O1—C7—N6—C6	−176.1 (3)
N3—C17—C18—C19	−177.2 (3)	C8—C7—N6—C6	2.5 (4)
C17—C18—C19—C20	−0.4 (6)	O1—C7—N6—C39	0.8 (6)
C18—C19—C20—C21	−0.5 (6)	C8—C7—N6—C39	179.4 (4)
C19—C20—C21—C22	0.4 (6)	O1—C7—N6—C39'	17.8 (15)
C20—C21—C22—C17	0.6 (5)	C8—C7—N6—C39'	−163.6 (14)
C18—C17—C22—C21	−1.4 (5)	C1—C6—N6—C7	1.4 (4)
N3—C17—C22—C21	177.1 (3)	C5—C6—N6—C7	−178.3 (4)
N4—C16—C23—C24	−51.8 (4)	C1—C6—N6—C39	−175.5 (5)
C14—C16—C23—C24	124.6 (4)	C5—C6—N6—C39	4.7 (7)
N4—C16—C23—C28	129.7 (3)	C1—C6—N6—C39'	165 (2)
C14—C16—C23—C28	−54.0 (5)	C5—C6—N6—C39'	−15 (2)
C28—C23—C24—C25	0.2 (6)	C11—C10—S1—C9	18.3 (3)
C16—C23—C24—C25	−178.4 (4)	N1—C9—S1—C10	4.0 (3)
C23—C24—C25—C26	0.0 (7)	C7—N6—C39—C40	97.6 (8)
C24—C25—C26—C27	0.1 (7)	C6—N6—C39—C40	−85.8 (9)
C25—C26—C27—C28	−0.5 (7)	C39'—N6—C39—C40	35 (8)
C26—C27—C28—C23	0.8 (6)	N6—C39—C40—C41	85 (2)
C24—C23—C28—C27	−0.7 (5)	C7—N6—C39'—C40'	−84 (4)
C16—C23—C28—C27	177.9 (3)	C6—N6—C39'—C40'	113 (3)
C38—C12—C29—O2	−125.3 (3)	C39—N6—C39'—C40'	41 (5)
C13—C12—C29—O2	−0.9 (4)	N6—C39'—C40'—C41'	108 (4)

### Hydrogen-bond geometry (Å, º)

**Table:**

*D*—H···*A*	*D*—H	H···*A*	*D*···*A*	*D*—H···*A*
N5—H5*A*···O1^i^	0.92 (4)	1.92 (4)	2.822 (4)	166 (3)

Symmetry code: (i) −*x*, −*y*, −*z*+1.
